# MRI for attenuation correction in PET: methods and challenges

**DOI:** 10.1007/s10334-012-0353-4

**Published:** 2012-11-21

**Authors:** Gudrun Wagenknecht, Hans-Jürgen Kaiser, Felix M. Mottaghy, Hans Herzog

**Affiliations:** 1 Central Institute for Electronics, Forschungszentrum Jülich, 52425 Jülich, Germany; 2 Department of Nuclear Medicine, University Hospital, RWTH Aachen University, Aachen, Germany; 3 Department of Nuclear Medicine, Maastricht University Medical Centre, Maastricht, The Netherlands; 4 Institute of Neurosciences and Medicine, Forschungszentrum Jülich, Jülich, Germany

**Keywords:** PET/MR, MR-based attenuation correction, Brain, Whole body, Coils, Truncation artefact

## Abstract

In current combined PET/MR systems, PET attenuation correction is based on MRI, since the small bore inside MRI systems and the strong magnetic field do not permit a rotating PET transmission source or a CT device to be integrated. Unlike CT measurements in PET/CT scanners, the MR signal is not directly correlated to tissue density and thus cannot be converted by a simple transformation of intensity values. Various approaches have been developed based on templates, atlas information, direct segmentation of T1-weighted MR images, or segmentation of images from special MR sequences. The advantages and disadvantages of these approaches as well as additional challenges will be discussed in this review.

## Introduction

**Fig. 1:**
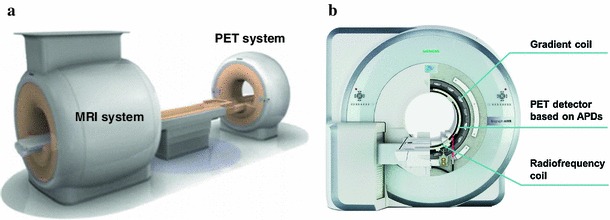
Hybrid PET/MR scanners: Philips Gemini TF PET/MR system where the PET and the MR system share a common patient table [1] (courtesy of H. Zaidi) (**a**); and Siemens mMR system where the PET detector is located between the gradient and the radiofrequency coils. (courtesy of R. Ladebeck and J. Georgi, Siemens Medical Solutions) (**b**)

The combination of magnetic resonance imaging (MRI) and positron emission tomography (PET) in hybrid systems has become a reality and such systems are currently being transformed from research prototypes into clinical systems. The system design provided by Philips is based on two separate gantries sharing a common patient examination table. This permits sequential data acquisition without repositioning of the patient between examinations to obtain spatially aligned image data (Fig. 1a) [1]. General Electric (GE) provides a tri-modality system with a transferable patient table which can be docked onto the PET/CT and MR system installed in two different examination rooms. The systems developed by Siemens for brain and whole-body imaging enable simultaneous acquisition of PET and MRI data since the PET scanner is fully integrated into the MRI system (Fig. 1b). All systems are based on standard clinical 3T MRI scanners. More information about the technical details and challenges of hybrid systems can be found in [2, 3].

Hybrid PET/MR systems provide complementary multimodal information about perfusion, metabolism, receptor status, and function, together with excellent high-contrast soft tissue visualisation without the need to expose the patient to additional radiation. Applications in neurology, psychiatry, and oncology from diagnosis to treatment planning and therapy control will benefit from multimodality measurements provided that technical problems will be overcome and fast examination protocols will be provided [4–6].

One of the most challenging issues of PET imaging in hybrid PET/MR systems is attenuation correction since the small bore inside MRI systems and the strong magnetic field do not allow PET transmission scans to be implemented with positron-emitting rod sources or additional computed tomography (CT) devices. Thus, present solutions for PET- or CT-based transmission systems are not compatible with the MR environment. Nevertheless, attenuation correction is indispensable for avoiding both qualitative and quantitative PET errors which could compromise diagnostic accuracy.

Thus, an ongoing research topic is the development of new MR-based attenuation correction approaches for brain and whole-body PET based on templates, atlas information, direct segmentation of T1-weighted MR images, or segmentation of images from special MR sequences. After introducing the topic of signal attenuation, the advantages and disadvantages of different MR-based attenuation correction methods and additional challenges will be presented and discussed in the remainder of the paper.

## Attenuation and PET/MR imaging

In order to obtain qualitatively and quantitatively accurate PET images, the emission data recorded during a PET scan do not only have to be reconstructed, but must also undergo different corrections. These corrections refer to normalisation for different detector efficiencies, random and scattered coincidences, dead time, decay, and, last but not least, tissue attenuation of the 511 keV photons which are emitted as pairs of opposing photons upon positron annihilation.

Photon attenuation is due to photoelectric interactions resulting in complete photon absorption or scattering with energy loss. The percentage of photons attenuated within the tissue is independent of the annihilation location, but dependent on the total intrabody travel length of the two 511 keV photons along a line-of-response (LOR) [7]. A length of, for example, 15 cm (medium diameter of the head) leads to an attenuation factor of 4.5, whereas a length of 35 cm such as found in the abdomen results in a factor of 18. Thus, only 22 and 5.5 %, respectively, of the radiation emitted by the radiolabelled tracer in the direction of an LOR is recorded by the PET detector. These numbers illustrate that even a minor error in measuring or determining the attenuation factor may lead to an erroneous correction for tissue attenuation.

Without any attenuation correction or with an erroneous correction, considerable regionally varying errors occur in the reconstructed PET images depending on the spatial distribution of tissue with different attenuation properties. In PET/MR, additional photon attenuation may be caused by coils located between the patient and the PET detector. Only if attenuation correction together with the other corrections indicated above is performed appropriately, is semiquantitative image analysis based on standard uptake values (SUV) feasible or further quantitative analysis [8] including kinetic modelling.

Attenuation correction can be performed in two different ways. One way is to pre-correct the measured emission data with the attenuation factors. These factors (attenuation correction factors (ACF)) can be derived from a transmission scan in PET-only scanners (nowadays practically obsolete, but still used in small animal PET) or by forward projecting the attenuation map (μ-map), which represents the spatial distribution of the attenuation coefficient, into sinograms. In PET/CT, the μ-map valid for PET is derived from diagnostic high-dose, contrast-enhanced or low-dose CT images by converting the Hounsfield units to μ values for 511 keV photons using piece-wise linear calibration curves [9, 10]. Before applying the conversion procedure, the CT images must be adapted to the PET resolution by Gaussian filtering and downsampling. The second method of correcting for tissue attenuation is to incorporate the knowledge on the μ-map directly into the iterative reconstruction as, for example, the 3D attenuation-weighted ordered subset expectation maximisation (OSEM) algorithm [11].

In hybrid scanners combining PET and MRI, it is not possible to derive μ-maps valid for PET from MR images using simple piece-wise linear calibration curves. Commonly, MR signals are related to the proton density and longitudinal (T1) and transverse (T2) magnetisation relaxation properties of the tissue under investigation, but they are not related to tissue attenuation in regard to ionising radiation. This becomes obvious with respect, for example, to bone and cavities which show similar signal intensities in MRI, but cause the highest and lowest tissue attenuation in PET.

Photon attenuation in PET/MR systems is due to the patient tissue itself and MRI system components such as the patient bed, immobilisation devices, and radiofrequency (RF) coils. In brain imaging, bone, air-filled cavities, and soft tissue are the most relevant classes for attenuation correction. In whole-body applications, lung tissue must also be taken into account [6], whereas bone may be regarded as less relevant as in brain imaging. Furthermore, the usable MR field of view (FOV) in present whole-body PET/MR scanners is too small to image the patient completely thus leading to truncation artefacts, which have to be considered in attenuation correction procedures.

## MR-based attenuation correction approaches

MR-based attenuation correction (AC) approaches consist of distinguishing the regions with different attenuation properties, assigning the correct linear attenuation coefficients to them and utilising the resultant attenuation map to correct the PET emission data during reconstruction. MR-based approaches were first developed for multimodal PET/MR acquisitions of the brain.

Multimodal brain and whole-body studies can be performed with hybrid whole-body PET/MR systems. Thus, the correction methods for brain data acquisition are also relevant for brain studies with whole-body PET/MR, especially because such methods are not presently available in whole-body PET/MR systems. The need has also arisen for additional MR-based attenuation correction approaches for whole-body applications, and some of the existing methods for brain imaging have been adapted for whole-body imaging. Other methods cannot be applied for the whole body because of the non-rigidity of the body, organs, and MR equipment which is particularly challenging. Four categories can be distinguished: template-based, atlas-based and direct segmentation approaches, and methods based on special bone-representing sequences.

### Template-based approaches

Template-based methods were initially suggested for situations where a transmission scan of the subject investigated is not available in PET [12]. The attenuation map template is constructed as an average image from a number of available transmission scans. In template-based methods utilising PET and MRI, an attenuation map template and a co-registered MR template are generated. After adapting the MR template to the patient MR image with nonlinear registration, the same nonlinear transformation can be applied to the attenuation map template to adapt it to the PET image of the patient investigated.

**Fig. 2:**
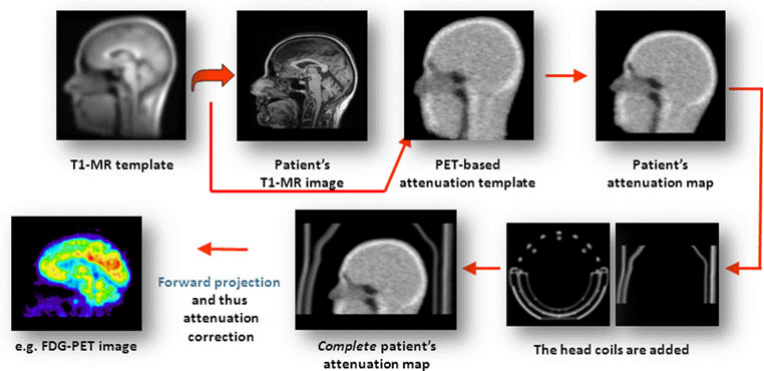
Steps in a template-based attenuation correction approach for brain [14]: The MR template is warped to match the individual MR image using SPM. The obtained transformation matrix is applied to warp the attenuation map template to generate an individualised attenuation map to which the coil attenuation map is added. The attenuation correction factors are obtained by forward projection

One such approach was presented by Rota Kops et al. [13, 14]. The average attenuation map template was generated from 68Ge-based transmission scans (HR+-PET) of 10 healthy subjects (females and males) via spatial normalisation to the standard brain of SPM2 [15]. Using the co-registered T1-weighted MR template of SPM2 for nonlinear registration with the MR image of the patient investigated, the transformation matrix obtained is applied to the attenuation map template to generate an individualised attenuation map [13]. In a second version, one of the measured image pairs is used as a reference instead of the SPM standard brain and the other data sets are nonlinearly registered to it [15]. In [14], separate female and male templates averaged over four volunteers each are generated. In the latest version, a mixed-gender template is constructed as an average of the eight subject data sets. Finally, the attenuation map of the MR head coil measured in the HR+-PET is added so that the method can be applied in the PET/MR scanner (Fig. 2).

Referring to work of Rota Kops et al., Malone et al. [16] composed their average template based on spatial normalisation of the individual MR images and the associated co-registered measured attenuation maps. For nonlinear registration, SPM2 or a B-spline free-form deformation algorithm [17] was used.

### Atlas-based approaches

Atlas-based approaches were developed to integrate global anatomical knowledge derived from a representative intensity-based or segmented reference data set into the segmentation procedure.

Schreibmann et al. [18] developed a multistep registration algorithm (rigid, B-spline, and optical flow) to deform one representative CT data set to match the individual patient MR image. This synthetic patient CT is then used for PET attenuation correction.

Hofmann et al. [19, 20] used a set of 17 MR atlas (T1-weighted spin-echo images) and co-registered high-dose CT atlas data sets (120 kVp, 285 mAs) to generate a pseudo-CT for a new patient MR data set. For this purpose, the MR atlas data sets are nonlinearly registered with the patient MR image and the same transformations are then applied to the CT atlas data sets. The pseudo-CT data set is constructed as a weighted sum from each co-registered CT atlas data set. Since registration can be locally imperfect, additional local information is taken from the patient’s MR data set. For each voxel of the MR data set, a surrounding patch is used to estimate the best CT value and thus the attenuation correction value using a support vector machine trained with MR-CT pairs of the atlas database.

Hofmann et al. [21] adapted their method to whole-body applications. They changed the registration method, the kernel function of the pattern recognition method, and added pre- and postprocessing steps. The MR-CT atlas database was constructed from 10 MR-CT whole-body patient data sets. Utilising the a priori assumptions of the atlas, the atlas part of the method improves the results in case of truncation or artefacts induced by metallic implants. On the other hand, the atlas cannot account for pathological regions such as tumour regions which are not part of the atlas. Applying the pattern recognition part of the approach, at least soft tissue attenuation values are assigned to these regions [21]. The same piecewise linear mapping methods as in PET/CT [9, 10] can be used to convert the pseudo-CT values into attenuation correction values.

For atlas-based attenuation correction, a brain atlas was constructed by Malone et al. [16] composed of the BrainWeb and Zubal digital phantoms [22, 23] and manually edited to include two classes: one for the sinuses and one for the ethmoidal air cells or nasal cavity. The same registration methods as for the template-based approach were applied for atlas registration with individual patient data. These authors noted that one reason for the slightly poorer results compared to their template approach (see above) could be that registration of a mean template image to a single subject image might be more reliable than that of the single subject atlas to another single subject image [16].

### Direct segmentation-based approaches

**Fig. 3:**
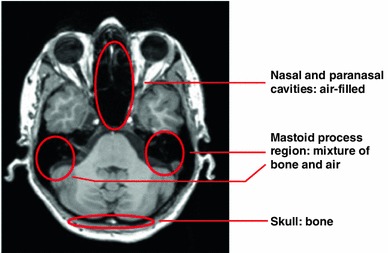
The T1-weighted MR image slice shows an air-filled nasal cavity, the mastoid process as a mixture of lamellar bone and small cavities, and the skull. The *dark areas* representing bone and air appear in the same intensity range

These approaches work directly on the standard T1-weighted MR images routinely acquired for each patient. The most challenging task in using these images is distinguishing bone tissue from air-filled cavities since both tissue types appear in the same intensity range (Fig. 3).

Thus, additional anatomical information, such as that skull encloses the brain and is covered by subcutaneous fat, must be utilised to distinguish these tissues. On the other hand, correct segmentation of these tissue types is crucial because of their different attenuation properties. Bone segmentation errors can thus lead to large biases in adjacent grey matter structures [13, 24].

Zaidi et al. [25] developed a segmentation approach based on fuzzy clustering to segment T1-weighted MR images into air, skull, brain tissue, and nasal sinuses further refined with morphological operations. Tissue-dependent attenuation coefficients were derived from [26]. Rota Kops et al. [13] used BrainSuite2 [27] and the MPITool (Advanced Tomo Vision GmbH, Kerpen, Germany) to distinguish bone, cavities, brain, and soft tissue to which the corresponding attenuation coefficients were assigned [28, 29]. They mentioned that distinguishing bone and cavities is the most demanding task, especially if the method is intended for clinical routine.

**Fig. 4:**
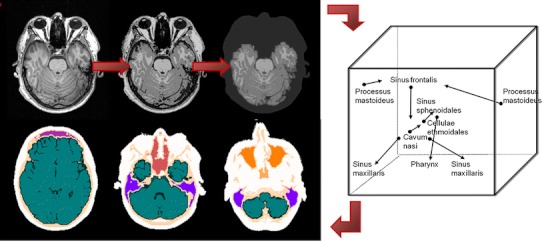
Principles of the direct knowledge-based segmentation approach for attenuation correction in brain studies presented in [31]: The input image (*top left*) is classified (*top middle*) and postprocessed to separate the extracerebral region (*top right*). The extracerebral region is segmented utilising class properties and relative positions of the regions (*right*). Segmented cavities and bone regions are the frontal sinus (*mauve*), the nasal cavity/ethmoidal cells/sphenoidal sinus (*dark red*), the maxillary sinuses (*orange*) and the pharynx (*amber*), the mastoid process (*light blue*), bone (*light pink*), brain (*green*) and CSF (*dark green*) tissue, and extracerebral soft tissue (*white*) (*bottom*)

A more sophisticated segmentation method was introduced by Wagenknecht et al. [30–33] making use of anatomical knowledge about the relative position of the regions to each other and the different shape together with tissue classification in an automatic multistep approach. Neural network-based tissue classification distinguishes grey and white matter, cerebrospinal fluid, adipose tissue, and background. Knowledge-based postprocessing separates the brain region from the extracerebral region and segments the extracerebral region. Regions of different shapes and sizes are detected with simple rectangular 2D patches in a fixed order to utilise the anatomical knowledge about the relative position of the regions and to change the membership of the tissue class for each voxel depending on those regions already segmented and classified. Ultimately, brain tissue, extracerebral soft tissue, the neurocranial and craniofacial bone, different air-filled craniofacial nasal and paranasal cavities, and the mastoid process in the temporal bone are distinguished. The mastoid process consists of lamellar bone and air-filled entities and thus is segmented as a separate region to enable the assignment of a special attenuation coefficient [30]. A preprocessing step correcting for inhomogeneities and a method reducing the fat shift artefact in 3T MR data were added to further improve the results [31] (Fig. 4).

Direct segmentation-based approaches were also developed for whole-body PET/MR, segmenting T1-weighted MR images into lung, fat, soft tissue, and background. Most of the approaches do not consider bone since, for example, chest and vertebral bones are not distinguishable in the MR images.

Hu et al. distinguished air, lungs, and soft tissue. Patient size and orientation is estimated by histogram analysis and intensity thresholding used for soft tissue segmentation. For lung segmentation, a combined intensity-based region growing and deformable model-based approach was developed to reduce leakage into the stomach and bowel. Anatomical knowledge, for example, about the typical position and size of lung to body, and morphological features, such as compactness, were utilised. Postprocessing with region growing including a distance constraint and additional morphological opening was added to further improve the lung segmentation [34, 35]. A similar method was reported by Zaidi et al. [1]. Schulz et al. [36] presented an approach based on Laplace-weighted histograms to threshold the outer body contour and to segment the lung by restricted region growing, making use of additional size criteria to identify the correct clusters. Additional 3D region growing using a relative threshold finally segments the lung compartment. Akbarzadeh et al. [37] segmented up to four classes—soft tissue, lung, spongious, and cortical bone—using the ITK library.

### Sequence-based approaches

Ultrashort echo time (UTE) sequences were developed to visualise anatomical regions such as tendons, ligaments, or bone having very short spin–spin relaxation times T2. Thus, UTE sequences are expected to differentiate bone from air. Therefore, UTE sequences may allow all attenuation-relevant regions to be distinguished solely on the basis of the MR image contrast without using any additional anatomical reference data.

UTE-based attenuation correction is based on MR acquisitions at two echo times. When both echoes are obtained during one acquisition, the sequence is called DUTE. The first image is obtained at TE1 (e.g. 70–150 μs [38]) measuring the sampled fast induction decay (FID) signal and visualises bone tissue. The second image is a gradient echo image at TE2 (e.g. 1.8 ms [38]), which does not show bone tissue. In both images, the signals of other tissues are similar. Keereman et al. suggested calculating a map of R2 values representing the inverse of the spin–spin relaxation time T2 from the signal intensities of these two images. The R2 map is used to segment cortical bone (R2 high) and soft tissue (R2 low). A binary mask created from the TE1 image by region growing and connected component analysis is used to mask and correct the R2 map for further distinguishing air and soft tissue. Finally, attenuation coefficients are assigned to the segmented regions [38, 39].

In a different approach reported by Catana et al. [24], soft tissue is masked with a morphologic closing and opening filter applied to the second echo data to segment the head and exclude voxels outside the head. The two echo images are divided by their smoothed versions to reduce inhomogeneities, and a normalised difference image is constructed to enhance the bone tissue before thresholding. A normalised additive image is used to threshold the air-filled cavities. All other head voxels are segmented as soft tissue. In a recent publication, Berker et al. [40] proposed a new UTE triple-echo (UTILE) MR sequence combining UTE for bone detection with Dixon water-fat separation [41] to distinguish four tissue classes (bone, air, soft, and adipose tissue) by postprocessing procedures. Attenuation maps were derived by assigning discrete attenuation coefficients to the classified voxels. For radiotherapy planning, Johansson et al. [42] developed a purely voxel-based method to generate a pseudo-CT, which they call substitute CT (s-CT). Using a Gaussian mixture regression model to train the MR-CT correspondences for three MR image types (two different UTE and one T2-weighted image) based on a number of patient data, the derived model was then used to generate an s-CT from the MR images of a new patient. The s-CT provides the attenuation coefficients on a continuous scale, such as in the method of Hofmann et al. [19, 20].

**Fig. 5:**
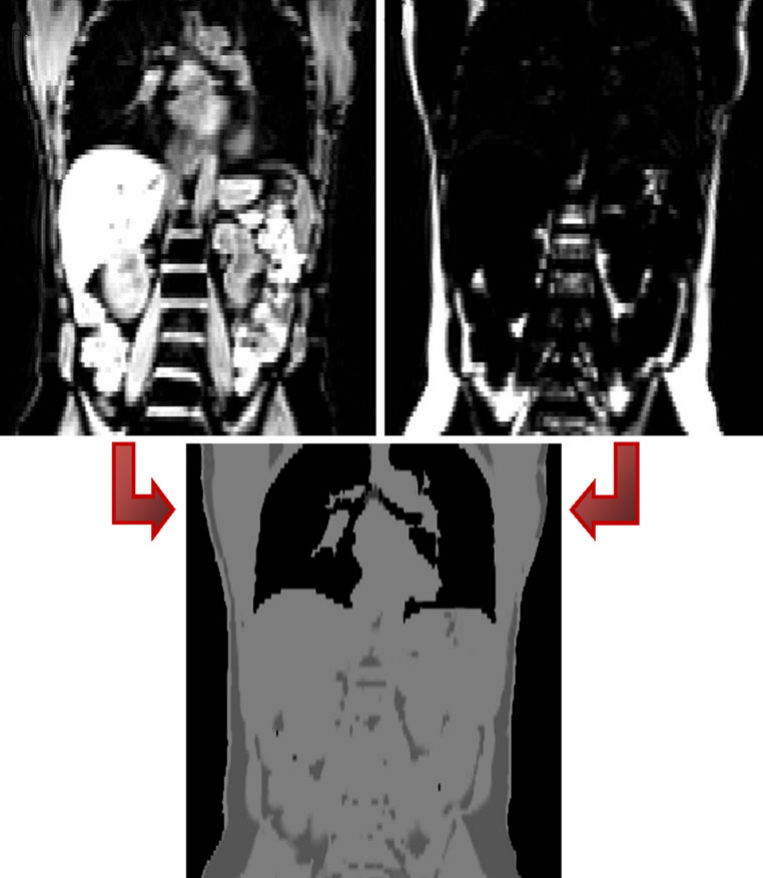
Dixon-based segmentation for whole-body attenuation correction shown in [44] (courtesy of A. Martinez-Moeller): MRI water (*top left*) and fat (*top right*) images acquired with a 2-point Dixon sequence are combined and segmented to generate the attenuation map for lungs, adipose tissue, soft tissue, and background

Two-point Dixon sequences [43], which need only a few seconds per bed position, provide separate images for water and fat and are thus well suited for the segmentation of whole-body MR images into lungs, adipose tissue, soft tissue, and background. Martinez-Möller et al. [44] proposed an automatic thresholding method for segmenting these tissue classes, which works on both images to separate soft tissue and fat from background regions. The lung regions were segmented as background regions inside the body by connected component analysis. Small misclassified regions were corrected by morphological closing. Bone tissue is thus not separated as an additional region but regarded as soft tissue (Fig. 5). Based on this approach, Hoffmann et al. [21] implemented the segmentation into air, lungs, fat tissue, fat–non-fat tissue mixture, and non-fat tissue utilising the additional in-phase image. This method combines thresholding within the different images and connected component analysis for lung segmentation.

## Advantages and disadvantages of current approaches

The most important advantages and disadvantages of current approaches are summarised in Table 1. Template-based methods depending on PET transmission scans are easy to use for brain applications. They are highly automated and robust and provide attenuation correction (AC) values on a continuous scale. Template–subject matching might be a problem in case of high anatomical variability, pathologies, and deformable organs or organ motion. Thus, these methods are less suitable for whole-body applications.

**Table 1:** Advantages and disadvantages of approaches for MR-based attenuation correction

	Brain	Whole body
Templates		
Pros	Robust Highly automated Continuous AC values	
Cons	Mapping template subject: anatomical variability, pathologies	Mapping template subject: non-rigid organs, organ motion Not suitable
References	[13, 14, 16]	
Atlases		
Pros	Robust Integration of local information overcomes problems with pathologies Continuous AC values (CT-based atlases)	Robust Accounts for truncation artefacts Integration of local information overcomes problems with pathologies Continuous AC values (CT-based atlases)
Cons	Atlas creation (CT, MR acquisition parameters) Mapping atlas subject: anatomical variability Computation time Discrete AC values (segmented atlases)	Atlas creation (CT, MR acquisition parameters) Mapping atlas subject: non-rigid organs, organ motion Computation time
References	[16, 18, 19]	[21]
Direct segmentation		
Pros	Individual patient data Reference data not needed No additional sequences Segmentation accuracy Computation time	Individual patient data Reference data not needed No additional sequences Segmentation accuracy Computation time
Cons	Robustness depends on anatomical assumptions Discrete AC coefficients	Robustness depends on anatomical assumptions Discrete AC coefficients
References	[13, 25, 30–33]	[1, 34–37]
Sequences		
Pros	Individual patient data Cortical bone visualised (UTE) Reference data not needed Segmentation accuracy Computation time	Individual patient data More differentiated tissue distribution (Dixon) Reference data not needed Segmentation accuracy Computation time
Cons	Additional acquisition time Discrete AC coefficients	Additional acquisition time Discrete AC coefficients
References	[24, 38–40, 42]	[44]

Approaches utilising atlases integrate global anatomical knowledge of an anatomical reference data set into the individual attenuation map. To reduce local imperfections due to registration failures, mixed atlas-classification-based approaches combine global and local information. First developed for brain applications, they have also been adapted to whole-body requirements. Since they are based on nonlinear registration of atlas and subject, the same problems as in template-based methods may occur. On the other hand, artefacts such as truncation can be overcome. Integration of local information improves the results in case of pathologies such as tumours which cannot be modelled with an atlas. Atlas generation depends on the underlying CT and MR acquisition parameters, and computation time is a problem in whole-body applications. Except for the Malone approach [16], attenuation values are predicted on a continuous scale.

Direct segmentation approaches can be used in brain and whole-body applications and work directly on the routinely acquired T1-weighted MR images of the patient investigated. Additional anatomical information about the shape and position is used to distinguish regions showing similar MR intensities, but different attenuation properties such as bone and cavities. They are, in principle, able to outperform nonlinear registration-based methods in segmentation accuracy and computation time and are more suitable in case of anatomical variability if as few assumptions as possible are made about normal anatomy. A disadvantage is the need for discrete attenuation coefficients for regions showing high interindividual variability of tissue density such as the lungs.

Accepting additional acquisition times, sequence-based approaches can be seen as a refinement of direct segmentation approaches, utilising the information provided by additional MR sequences to represent bone (e.g. UTE) or fat and water (Dixon) to improve the segmentation of attenuation-relevant regions in the brain and whole body.

## Challenges of MR-based attenuation correction

Further problems with accurate attenuation correction remain to be solved [5] to avoid errors in the MR-derived attenuation maps and their propagation to the reconstructed PET emission images.

### Assignment of attenuation values

Whereas continuous attenuation values represent the variable density of body tissue, discrete attenuation coefficients are used for segmented MR-based attenuation correction. Thus, analysing the interindividual variability of tissue density and its influence on attenuation correction in PET is an important issue for all MR-based attenuation correction approaches using predefined attenuation coefficients [32].

Regions showing a large interpatient variability of attenuation values are the lung region consisting of millions of alveoli filled with air [44], bone consisting of hydroxyapatite, collagen and water [45], and other unpredictable benign or malignant abnormalities with varying density across patients [46]. Interpatient variability of attenuation coefficients can lead to non-negligible errors in the PET emission data. Particularly, lung density shows a high degree of interpatient variability of up to 30 %, depending on age, disease, and breathing patterns [7, 46]. Keereman et al. [6] estimated errors of 10 % and more in their study due to interpatient variability of lung attenuation coefficients. Therefore, they suggested analysing whether individual properties such as age, constitution, and smoking habits can be used to predict lung density.

Schulz et al. [36] used patient-individual versus predefined attenuation coefficients. In comparison with CT-based attenuation correction, they found the largest difference in SUV for bone lesions when using predefined attenuation coefficients. Schleyer et al. [47] investigated different constant patient-specific and generic attenuation coefficients for bone in whole-body imaging. Using patient-specific mean bone values, the maximum average error in the lung region was 5 %, whereas an overestimated bone volume with generic soft tissue values produced an error of 10 % in the lung. They conclude that segmenting multiple bone compartments would improve the attenuation-corrected PET images.

Besides the actual interpatient variability of attenuation values, the dependence on the number of discriminated classes and the segmentation accuracy are important issues in segmentation-based approaches. Keereman et al. [38] analysed the influence of chosen attenuation coefficients as well as misclassification errors in lung and different bone compartments for whole-body applications. They found that the lung tissue must be considered as a separate region because it represents a large area with different attenuation property. In accordance with the results reported by Samarin for bone lesions [48], quantitative PET results obtained by Keereman et al. [39] showed that bone segmentation is mandatory since confusing bone with soft tissue has a great effect on reconstructed SUV. Confusing bone with air can result in erroneous lesion and tumour detection. Errors in the reconstructed PET images grew from less than 5 to 17 % when bone tissue was ignored in the attenuation map and up to 45 % when lung tissue was ignored [6]. Keereman et al. concluded that at least air, lung, soft tissue, spongious bone and cortical bone and, if possible, adipose tissue should be distinguished for attenuation correction ensuring errors remain below 5 %. In addition, they noted that in small animals bone structures are much smaller than in humans and segmentation errors or even completely disregarding the bone would therefore not introduce as large errors as in humans. Akbarzadeh et al. [37] attempted to quantify how many classes are needed to minimise the error induced by missing classes and underestimated SUV by 11 %, neglecting the bone class. Furthermore, they found that the global error increases with decreasing numbers of classes. 

The definition of correct attenuation coefficients is a further challenge. Table 2 summarises the attenuation coefficients used by different groups for brain and whole-body applications.

**Table 2:** Attenuation coefficients used in brain and whole-body applications in the indicated references (units in 1/cm)

Brain	Whole body
Air/cavities/background	
0.0 [13, 24, 25, 30–33, 38] 0.003 [40] 0.0536 (nasal sinuses) [25] 0.054 (mastoid process) [30–33] 0.000105 (air, paranasal sinuses), 0.066 (ethmoidal air cells, nasal cavity) [16]	0.0 [21, 36, 44, 45, 49]
Skull/bone	
0.116 [40] 0.12 [38] 0.143, 0.151 [24, 25] 0.146 [13, 30–33] 0.143, 0.152, 0.172 [16]	0.11 (spongious bone) [6] 0.13 [6, 45] 0.12–0.14 [47] 0.15 [49]
Soft tissue	
0.086 (soft), 0.064 (fat) [40] 0.095 [13, 30–33] 0.1 (muscle, skin, connective tissue), 0.092 (fat) [16]	0.093, 0.086 (fat) [21] 0.093 (fat–non-fat), 0.101 (non-fat) [21] 0.094, 0.086 (fat) [36] 0.095 [47] 0.0968 (soft), 0.0927 (fat) [6] 0.097 [45] 0.1, 0.086 (fat) [44, 49]
Brain tissue	
0.095 [38] 0.096 [24, 30, 33] 0.099 [13, 31, 32] 0.0993 [25] 0.097 (CSF), 0.1 (GM,WM) [16]	
Lung tissue	
	0.018 [44] 0.024 [21, 36] 0.0267 [6] 0.03 [45, 49]
Liver tissue	
	0.0977 [6]

### Image reconstruction and registration

The reconstruction method used to obtain the final PET emission images also influences the final result. Iterative reconstruction methods such as 3D OSEM differ in the implementation and parameters used (e.g. subsets, number of iterations) and yield emission images with different matrices and voxel sizes. Furthermore, scatter correction as part of the reconstruction process utilises the attenuation map and may disturb the reconstructed results if the map is prone to error. Such errors may be related to segmentation and registration problems or truncation artefacts leading to erroneous scatter correction scaling.

Misregistration between the emission image and the attenuation map must be avoided during reconstruction, since even small emission-transmission misregistration can influence the final result. Another error source is patient or organ motion, such as cardiac and respiratory motion as well as blood flow, which can cause motion artefacts in the MR images and thus tissue misclassification and/or registration errors. Respiratory- and cardiac-gated MRI can lead to mismatches for MR-based attenuation correction. Whereas Keereman et al. [6] suggested using the average attenuation over the whole respiratory cycle to avoid the mismatch, Hofmann et al. [20] regarded 4D MR-based attenuation correction as a great advantage over CT-based attenuation correction. Buerger et al. [49] presented a 4D approach, combining a respiratory-gated UTE sequence and a short dynamic MR sequence. The segmented 4D attenuation map was derived from both acquisitions and used for attenuation correction of multiple respiratory PET gates. Hofmann et al. [20] also noted that further local mismatches can occur which cannot be corrected and, as an example, mentioned local misregistration due to pockets of gas in the abdomen. Furthermore, especially for atlas- and template-based methods, the influence of the chosen registration approaches on the final results is not negligible. This is shown in [16] for the author’s approach comparing B-spline and SPM2-based registration.

### Coils and other devices

**Fig. 6:**
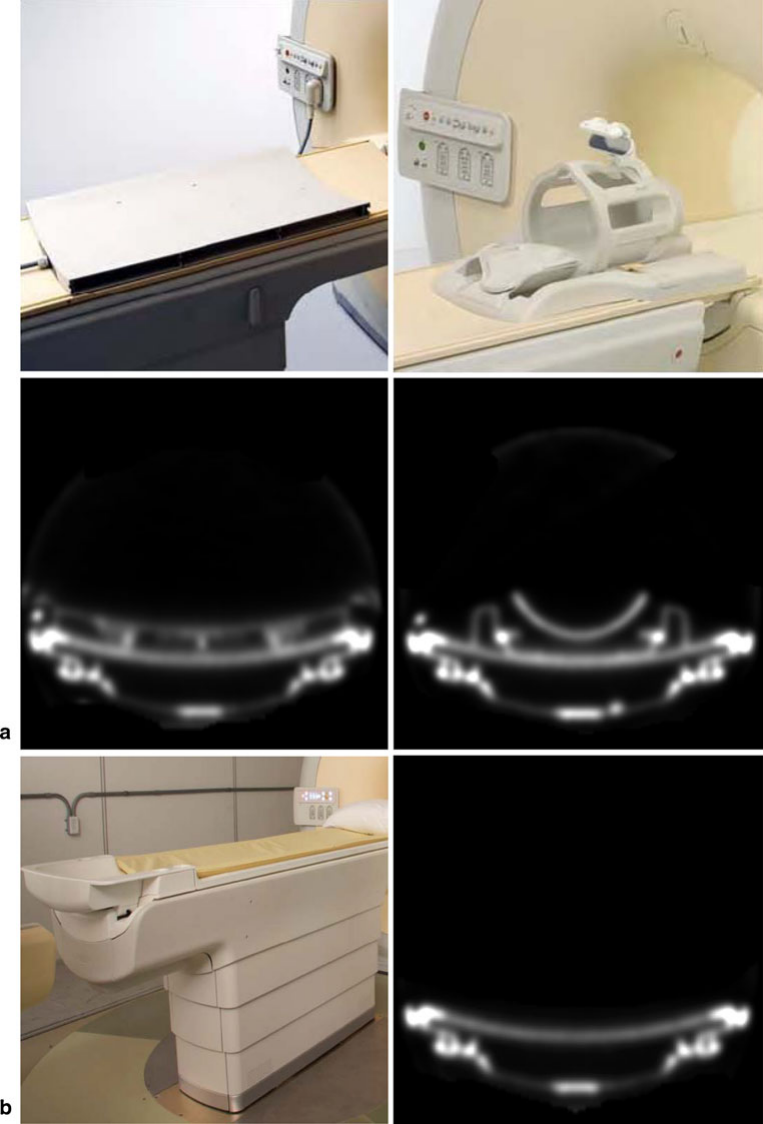
In PET/MR, coils and other MR devices not visible in the MR images must be generated as template images, for example, in PET transmission scans and added to the attenuation map. Examples of a spine and a head coil (**a**) and a patient table (**b**) as well as one transaxial slice of the corresponding templates generated from PET transmission scans are shown [54] (courtesy of B. Zhang)

Coils and other MR devices of high-density materials inside the PET FOV (e.g. examination table, patient positioning and immobilisation devices, headphones, prostheses and implants, medical probes (Fig. 6)) are not visible in MR images but contribute considerably to the attenuation map [46]. They must therefore be added to the attenuation map in the correct spatial location [44], since inaccurate positioning will otherwise become a relevant source of error [38]. Ignoring the coil attenuation in brain PET can lead to errors of up to 50 % [24]. Coils can be measured in CT and, after rescaling the Hounsfield values to 511 keV attenuation coefficients, added to the attenuation map. This can be easily done for rigid head coils, but presents a problem for deformable coils such as flexible surface coils for whole-body applications. Thus, specially designed RF coils with lower attenuation and positioning strategies based on coil landmarks must be developed to reduce these problems [20, 50].

Mantlik et al. [51] analysed the deviation of PET activity values when positioning devices were disregarded for attenuation correction. They found a regionally variable distortion yielding an overall underestimation of activity concentration (8.4 % for DOTATOC, 7.4 % for FDG images) for head/neck patient data and an insignificant decrease for the lower extremities. Delso et al. [52] found that head and neck coils induce 17 % count loss in PET transmission scans and that the misregistration of coil templates should not be greater than 1–2 mm in the axial direction to avoid unacceptable image artefacts. The medical probes tested in this study had only a local influence and thus should be positioned as far as possible from the regions of interest. On the basis of phantom experiments, Tellmann et al. [53] showed that dismissing MR surface coil attenuation causes a bias in regions near the coils and small objects in central regions. Coil misalignment of several cm between emission and attenuation images led to errors comparable to those of unaccounted MR coil attenuation.

Zhang et al. [54] concluded that coil templates generated from PET transmission scans are more accurate and artefact-free compared to those generated from CT scans (Fig. 6). Also Hu et al. [34] suggested scanning additional devices in PET rather than in CT to create attenuation templates, since the conversion from CT to PET energy is not validated for non-biological materials. Furthermore, the CT images of the coils must be filtered so that they match the resolution of the PET. MacDonald et al. [55] measured an underestimation of 19 % when the head coil is not included in the attenuation map, but a 28 % overestimation due to the overestimated high-density components within the coil in the CT. They suggested redesigning the coils by rearranging the most attenuating materials. Herrick et al. [56] mentioned that due to the increasing relative size of MR hardware compared to the size of the animal measured in preclinical applications, and the resultant attenuation effects may exceed that of soft tissue or bone.

### Truncation artefacts

**Fig. 7:**
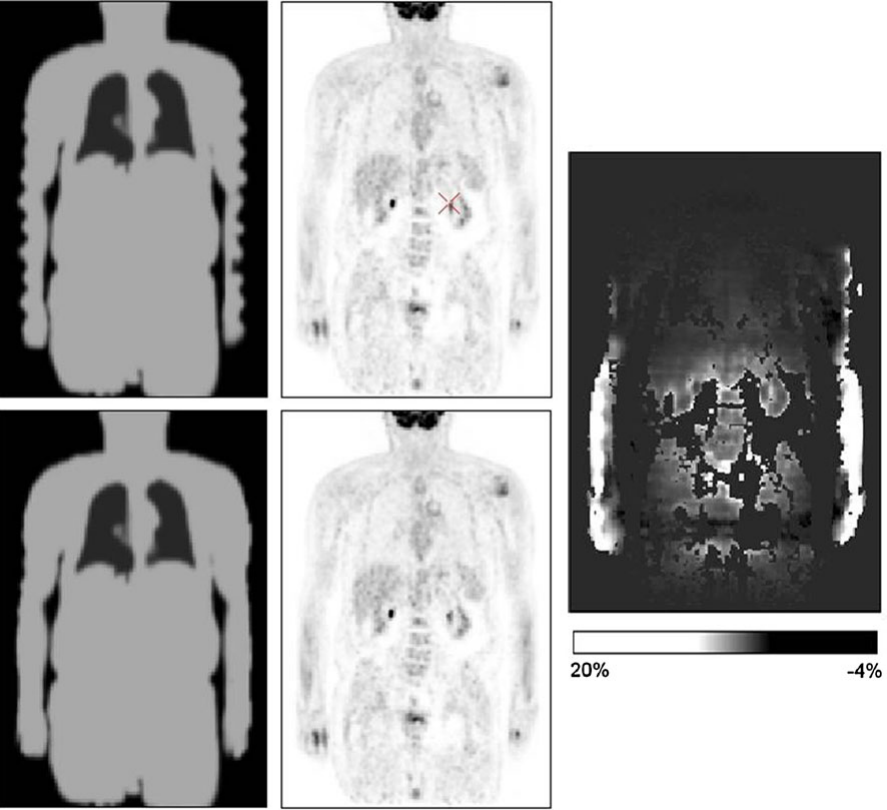
The arms are not completely covered in the whole-body MR scan leading to truncation artefacts in the attenuation map image (*top left*), which were successfully compensated (*bottom left*). The corresponding reconstructed PET images are shown in the *middle column*. The relative percentage difference image (*right*) shows the greatest changes around the arms, and moderate changes inside the trunk with the highest changes observed at surfaces of anatomical structures [35] (courtesy of Z. Hu)

Structures in the PET FOV which are truncated due to the limited FOV of clinical MRI systems are a further challenge. The small bore of MRI systems and additional coil equipment makes it uncomfortable for the patient to be scanned with raised arms as in PET/CT scanners. For patients scanned with arms down in hybrid PET/MR systems, the arms are often not completely covered in the whole-body MR scans [44]. Thus, truncation artefacts depend on how the patient is positioned and can also occur for other parts of the body, for example, breasts or hips. Delso et al. [57] analysed the bias introduced by an incomplete attenuation map on the basis of simulated and clinical data and found average biases of up to 15 %, which could be reduced to less than 10 % by adding the missing parts based on a 3D snake algorithm, which outlines the patient’s body in the non-attenuation-corrected PET data. A similar method based on edge detection in PET emission images and the assignment of soft tissue attenuation to the MR-truncated part was presented by Hu et al. [34, 35] (Fig. 7). Tang et al. [58] evaluated an improved method with the NCAT phantom using Monte Carlo (MC) simulations. They reduced the relative errors of up to more than 50 % resulting from truncation to less than 10 %. Nuyts et al. [59] developed a modified iterative maximum likelihood reconstruction of attenuation and activity (MLAA), which estimates the truncated part of the attenuation map from the PET emission data. The evaluation showed that the shape of the truncated arms could be well reconstructed. Thus, the method seems to be suitable for the completion of MR-based attenuation maps. Recently, Defrise et al. [60] suggested a new method which aims to reconstruct both attenuation and activity images from PET emission data alone, if these data are acquired with a time-of-flight (TOF) PET scanner. This may overcome the problem of body parts that are not seen in the MR image.

### Evaluation

In PET, the measured value in a single voxel is affected by the signal attenuation of all voxels on all lines-of-response (LORs) intersecting with this voxel. Thus, errors in the attenuation map do not only have a local effect on the reconstructed PET image [38]. Two different evaluation methods should be applied to assess and compare MR-based attenuation correction methods.

First, the MR-based attenuation map and/or the segmented MR image should be compared to a gold standard image, for example, a CT image, and local errors should be examined. This can be done on a voxel-by-voxel basis by analysing the difference image. In the case of segmented data, the Dice coefficient can be used, which represents the overlap of two segmentations. Second, the resultant attenuation-corrected PET image should be compared to a reference attenuation-corrected PET image, for example, obtained with CT-based attenuation correction. Differences can be studied on a voxel-by-voxel basis or in certain regions or volumes of interest by calculating percentage-relative differences of the resultant emission values. Maximum or mean absolute differences are appropriate quality measures, whereas mean differences show only the overall bias since positive and negative values may compensate each other [20].

Besides the intrinsic limitations of using CT-based attenuation correction as a gold standard (e.g. different energy, resolution, and artefacts), the sample size is a general problem in evaluating MR-based versus CT-based attenuation correction because images of the same patient from MRI, CT, and PET are rare. Thus, most of the methods were evaluated on different clinical data sets available at the respective sites. Small sample sizes can lead to problems in the significance of statistical results [19]. Tri-modality systems could help to resolve the sample size issue. Final comparisons therefore remain to be made and conclusions to be drawn in future. New phantoms simulating the attenuation properties of the most important tissues may help in doing so [45].

One of the most important evaluation issues is the question of how large attenuation correction errors may be without influencing the clinical diagnosis. If errors in the emission image show large spatial variations due to spatially varying errors in the attenuation map, the influence on diagnosis depends on the organ examined and the region used as a reference (e.g. the cerebellum in brain studies). For their data, Malone et al. [16] found the highest variability in parts of the frontal cortex near the sinus regions, which may reduce the detection of significant changes between groups or subjects, for example, in frontotemporal dementia. Hofmann et al. [21] predicted that the limit is an error of 10 %, and smaller errors do not influence diagnosis, particularly in oncology. The answer depends on the disease, the organ examined, and the clinical indication. Due to the increasing number of whole-body PET/MR systems, clinical studies become possible that allow the importance of quantitative accuracy versus that of reproducibility to be determined [36, 61]. A further challenge remains automation in patients with large deviations from normal anatomy. Thus, in general, the choice of method depends on the accuracy needed in clinical routine as well as the robustness and the computation time required for its execution [20].

## Summary

This paper gives a short introduction to the physical problem of attenuation in PET and the special features in PET/MR. We reviewed the different MR-based approaches for attenuation correction in PET/MR imaging of the brain and the whole body. Each of the reviewed methods has some inherent advantages and disadvantages regarding robustness, accuracy, and effort. Further challenges associated with MR-based attenuation correction methods were presented, including the appropriate assignment of attenuation values, the influence of image reconstruction and registration, the handling of coils and other MR devices, the correction of truncation artefacts, and the appropriate evaluation. These issues have to be taken into account to improve attenuation correction methods for future use in routine clinical applications.
